# Deformation Characteristics, Formability and Springback Control of Titanium Alloy Sheet at Room Temperature: A Review

**DOI:** 10.3390/ma15165586

**Published:** 2022-08-15

**Authors:** Hao Li, Shuai-Feng Chen, Shi-Hong Zhang, Yong Xu, Hong-Wu Song

**Affiliations:** 1 Shi-changxu Innovation Center for Advanced Materials, Institute of Metal Research, Chinese Academy of Sciences, Shenyang 110016, China; 2 School of Materials Science and Engineering, University of Science and Technology of China, Shenyang 110016, China

**Keywords:** titanium alloy, room temperature, mechanical properties, formability, springback

## Abstract

Titanium alloy sheets present inferior formability and severe springback in conventional forming processes at room temperature which greatly restrict their applications in complex-shaped components. In this paper, deformation characteristics and formability and springback behaviors of titanium alloy sheet at room temperature are systematically reviewed. Firstly, deformation characteristics of titanium alloys at room temperature are discussed, and formability improvement under high-rate forming and other methods are summarized, especially the impacting hydroforming developed by us. Then, the main advances in springback prediction and control are outlined, including the advanced constitutive models as well as the optimization of processing paths and parameters. More importantly, notable springback reduction is observed with high strain rate forming methods. Finally, potential investigation prospects for the precise forming of titanium alloy sheet in the future are suggested.

## 1. Introduction

Titanium and its alloys have the advantages of low density, high strength, high temperature resistance and corrosion resistance, which have advantages in engineering applications in the fields of aerospace, maritime, medical apparatus, instruments, etc. [1,2,3]. Specifically, titanium alloy is preferred for the manufacture of some critical thin-walled components utilized in aerospace due to its excellent mechanical characteristics. The application of titanium alloys provides excellent service performance and ensures long service life. Typical products made from titanium alloys are aircraft and aerospace engines. Although titanium alloy has many advantages in the manufacture of critical aerospace components, it is very difficult to obtain satisfactory geometric accuracy after forming due to its poor formability and severe springback at room temperature [4], which has brought tremendous challenges to traditional forming technologies. Therefore, it is necessary to propose novel forming methods to resolve this production problem with titanium alloys.

According to recent investigation reports, the plasticity of titanium alloy can be increased by superplastic deformation at low strain-rates (10^−5^ s^−1^~10^−4^ s^−1^), but the plasticity improvement relies on specific grain sizes of materials and high deformation temperatures [5]. Additionally, hot-forming of titanium alloy sheets at common strain rates (10^−3^ s^−1^~10^2^ s^−1^) is another feasible approach to improve the formability of titanium alloys [6,7,8,9]. To reduce unexpected springback of titanium alloy components, thermal creep forming (10^−4^ s^−1^~10^−2^ s^−1^) [10], electropulse forming [11] and electromagnetic pulse-assisted calibration methods [12] were developed. However, these forming methods are combined with high heating temperatures [13,14] and several forming stages [15], which induce high manufacturing costs and low forming efficiency. Specifically, when titanium alloys are exposed to high-energy fields for prolonged periods, they may produce coarse grain [16,17], oxidation [18] and hydrogen absorption [19,20] in the microstructures, which often cause forming defects and decreased application life. Therefore, it is difficult to simultaneously achieve both high forming efficiency and accuracy with the abovementioned forming processes. However, it is still important to develop a feasible forming method to achieve the increase in forming efficiency and accuracy for titanium alloy sheets.

It should be noted that many scholars have found that high strain-rate loading can effectively promote an increase in plasticity in lightweight alloys [21,22]. Hence, forming technologies, such as electromagnetic forming (EMF) [23,24], electrohydraulic forming (EHF) [25,26], explosive forming [27], impact hydroforming (IHF) [28], etc., coupled with high strain rates have been gradually developed in recent years [29]. Corresponding investigations are mainly focused on the deformation and springback behaviors of lightweight alloy sheets under EMF and IHF at room temperature [30,31]. It has been preliminarily proven that the formability of lightweight alloy sheets could be increased and the springback amount could be significantly reduced by controlling forming velocities within a reasonable range [32]. The abovementioned forming technologies provide one type of novel solution for the precise forming of titanium alloy sheets at room temperature. Specifically, the forming efficiency will be largely improved with these novel forming methods. However, the potential macro- and micro-mechanisms of formability improvement and springback reduction under high strain-rate loading have not been clearly presented, which limits the further application of high strain-rate forming.

In this paper, recent research on the mechanical behaviors and microstructure evolution of titanium alloy sheets under a wide range of strain-rate loadings were reviewed. Specifically, the high strain-rate forming technologies on titanium alloy and other lightweight alloys were explored. To understand the advantages of this forming technology, the formability of titanium alloys at different strain rates have been analyzed and compared. Additionally, the earlier investigations of IHF technology on lightweight alloy sheet forming developed by our research team were highlighted. Possible mechanisms for formability improvement and springback reduction in titanium alloys at high strain rates are inferred based on the existing literature and our IHF experimental results. Finally, suggested investigation topics and potential application of IHF on other lightweight alloys are summarized.

## 2. Deformation Characteristics of Titanium Alloys

Because of the poor plasticity of titanium alloys at room temperature, they are always used in hot forming processes. Therefore, most investigations are focused on the mechanical behaviors of titanium alloys at high temperature. However, many advanced forming methods for titanium alloys at room temperature have been developed in recent years; it is also important to summarize the deformation characteristics of them within a wide range of strain rates at room temperature. Similar to other metal alloys, the classification of mechanical characteristics of titanium alloys at room temperature mainly focuses on the strain/stress behaviors and formability (forming limit). According to current reports, the strain rate effect of titanium alloys (e.g., Ti-6Al-4V [33], Ti-8Al-1Mo-1V [34], Ti-10V-2Fe-3Al [35], etc.) at room temperature cannot be neglected and will have a significant effect on the forming results of titanium alloys. Considering that different titanium alloys contain different phase contents and types (α and β phase), the variation law in strain-stress behaviors under different strain rate loadings show important differences to each other at room temperature, especially between single-phase alloys (CP-Ti) and dual-phase alloys (Ti-6Al-4V alloy). Furthermore, some typical studies of the mechanical characteristics of titanium alloys have been introduced.

### 2.1. Strain and Stress Behaviors at Room Temperature

Classification of the mechanical characteristics of metals, including titanium alloys, under different loading modes is reflected by stress and strain. Among them, fracture strain can play a significant role in the plasticity of metal materials, while flow stress can reflect the strength of the metal. Therefore, the exploration of the variation law of the two parameters is beneficial to instruct the design of forming methods for titanium alloy components.

**Figure 1:**
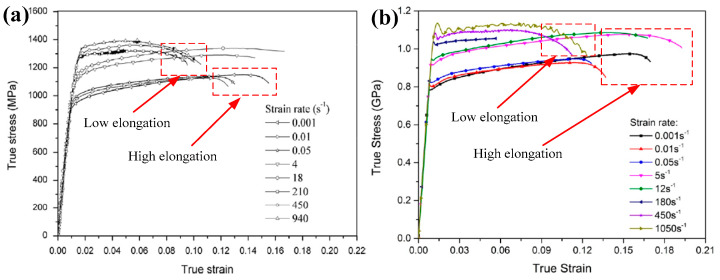
Tensile stress-strain curves of (**a**) Ti-6.6Al-3.3Mo-1.8Zr-0.29Si (TC11) alloy (Reprinted with permission from Ref. [38]. Copyright 2014, Mater. Lett.) and (**b**) Ti-5Al-2.5Sn alloy (Reprinted with permission from Ref. [21]. Copyright 2019, Materials.) in a wide range of strain rates at room temperature.

**Figure 2:**
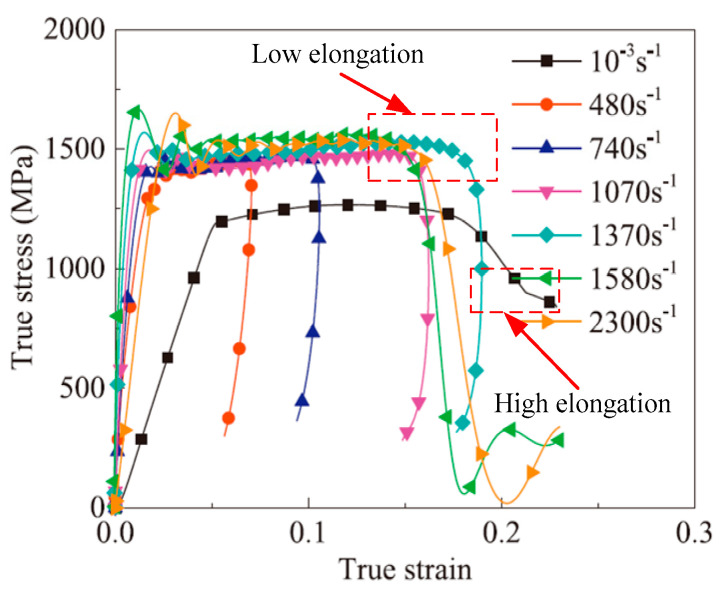
Compression stress-strain curves of Ti-55511 alloy in a wide range of strain rates at room temperature (Reprinted with permission from Ref. [39]. Copyright 2018, Mech. Mater.).

**Figure 3:**
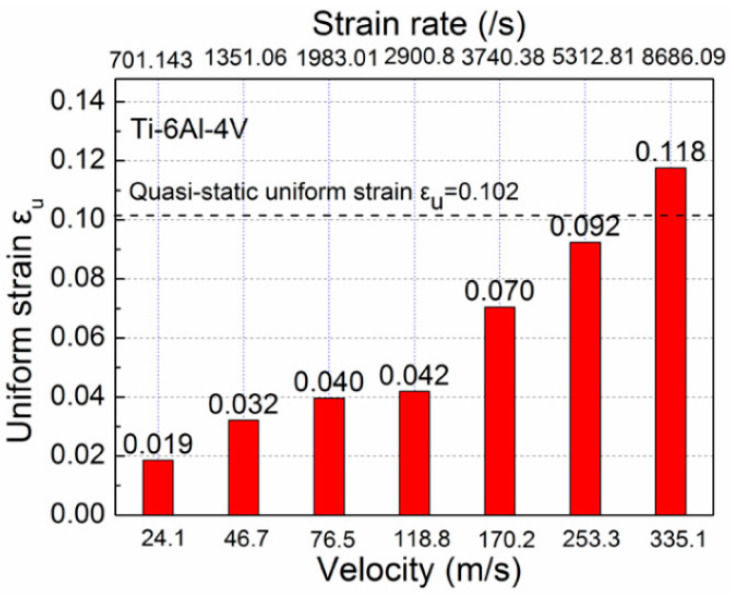
Relationship between strain rates, deformation velocities and uniform strain of Ti-6Al-4V alloy rings obtained from electromagnetic bulging experiments (Reprinted with permission from Ref. [40]. Copyright 2014, 11th International Conference on Technology of Plasticity).

As strain rate is known to affect the mechanical characteristics of materials, the strain rate effect on fracture strain under tensile loading is cause for concern. Due to the distinctive micro-structure of different titanium alloys, their fracture strain will present differently at different loaded strain rates. Many scholars have paid attention to this point and have acquired some valuable results. Tang et al. [36] found that the plasticity of Ti-10V-2Fe-3Al alloy changes slightly with the increase in loaded strain rates (3.33 × 10^−4^~1.2 × 10^3^ s^−1^), and the fracture strain increases slightly under high strain rate loading at room temperature. Harmmer et al. [37] investigated the stress and strain relationship of Ti-6Al-4V alloy under uniaxial tensile, compressive and shear loadings in a wide range of strain rates (1 × 10^−4^~1.5 × 10^3^ s^−1^) at room temperature and found that the fracture strain under the loaded high strain rates (>500 s^−1^) were all significantly lower than that under quasi-static loadings. Interestingly, similar concepts applied in the investigation of the responses of Ti-6.6Al-3.3Mo-1.8Zr-0.29Si (TC11) alloy (strain rates ranging from 10^−3^~940 s^−1^) and Ti-5Al-2.5Sn alloy (strain rates ranging from 10^−3^~1050 s^−1^) by Wang et. al. [21,38]. Their experimental results are shown in Figure 1. Similar to the stress-strain curves of Ti-6Al-4V alloy obtained under tensile loading, the fracture strain also initially decreased and then increased under compression loading at high strain rates (up to 3879 s^−1^). According to the above results, it was also found that the elongation of single-phase titanium alloys was larger than that of dual-phase titanium alloys at the same loaded strain rate. In contrast to the strain hardening behaviors of single-phase titanium alloys, stress drop occurs at the initial stage of plastic deformation to dual-phase alloys. Ran et al. investigated the compression stress-strain relationships of Ti-5Al-5Mo-5V-1Cr-1Fe (Ti-55511) alloy at a wide range of strain rates (480~2300 s^−1^); they also found that the elongation decreased under high strain rate loadings compared with quasi-static loadings [39], as shown in Figure 2. Li et al. [40] implemented electromagnetic bulging tests with Ti-6Al-4V alloy rings under the loaded strain rates of 1000~9000 s^−1^ and found that the obtained uniform strain and fracture strain were both significantly increased as the loaded strain rates were greater than 6935.6 s^−1^, as shown in Figure 3. Hence, the uniform elongation of Ti-6Al-4V alloy at high strain rate loadings exceeds that under quasi-static loadings only when the loaded strain rates are larger than 6935 s^−1^. Based on the above experimental results, the plasticity of titanium alloys will not always increase under high strain rate loadings until the loaded strain rates exceed one threshold value at room temperature referring to the quasi-static loadings. Therefore, determining the threshold value of the strain rate for plasticity improvement in titanium alloys at room temperature is worthy of further investigation.

**Figure 4:**
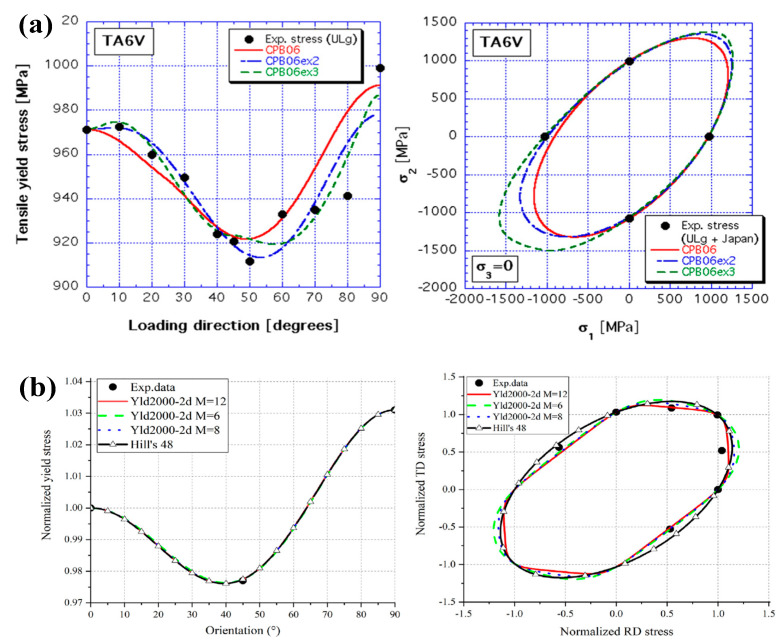
Yield stress and yield loci of Ti-6Al-4V under quasi-static tensile loadings (**a**) at strain rate 3.1 × 10^−4^ s^−1^ (Reprinted with permission from Ref. [42]. Copyright 2011, Int. J. Solids Struct.) and (**b**) at strain rate 0.1 s^−1^ (Reprinted with permission from Ref. [43]. Copyright 2020, Int. J. Solids Struct.).

Current investigations of flow stress behaviors of titanium alloys at different strain rates mainly focus on the variation law of yield and tensile strength. Considering the titanium alloys with hexagonal closed-packed phases, the yielding and hardening behaviors show anisotropic characteristics within different strain rates at room temperature [34,41]. Gilles et al. conducted a uniaxial tensile test of Ti-6Al-4V alloy at room temperature and obtained the yield stress in different loading directions and the corresponding yield loci at a strain rate of 3.1 × 10^−4^ s^−1^ [42], as shown in Figure 4a. It was found that the yield stress will initially increase and then decrease in the range of 45 degrees. Tang et al. reported that the yield stress presents a monotonic decrease with an increase in the directional degrees under tension at higher loaded strain rate of 0.1 s^−1^ [43]. Furthermore, the yield loci at different loaded strain rates has been reported to enhance the symmetry of ellipse at higher strain rates, as shown in Figure 4b. In contrast to the yield behaviors of titanium alloys, Zhang et al. discovered that the initial yield stress would increase with the increase in strain rates through the implementation of uniaxial tensile tests of Ti-5Al-2.5Sn alloy within the rate range of 10^−3^~10^1^ s^−1^ [21]; the stress-strain curves are shown in Figure 1b. Additionally, Zhang et al. found that the strain rate sensitivity increased at high strain rates. To investigate the hardening behaviors of titanium alloys at room temperature, Ran et al. implemented a series of dynamic compression tests for Ti-5Al-5Mo-5V-1Cr-1Fe (Ti-55511) alloy within the rate range of 10^−3^~2300 s^−1^ and discovered that the strain rate effect was enhanced under high strain rate loadings (Figure 2). However, according to the experimental results of Macdougall et al. within the rate range of 7 × 10^−4^~1000 s^−1^, strain rate hardening was apparent in tensile and shear loading to thin-walled tubular specimens of Ti-6Al-4V alloy [33]. Whereas, strain rate effect is poor under the compression loading condition at high strain rates at room temperature. That is, these results suggest that different titanium alloys may have quite different hardening behaviors under high strain rate loadings, which warrants further investigation in future work.

In our own investigations, the stress-strain behaviors of Ti-6Al-4V alloy sheet at rolling directions under a wide range of strain rates were investigated using a split Hopkinson tensile bar (SHTB) device. The results show that the elongation of Ti-6Al-4V alloy increases when the loaded strain rates are over 3000 s^−1^, the increased magnitude of which can be up to nearly 20% at a rate of around 4000 s^−^^1^. Therefore, it can be inferred that a certain range of high strain rates can cause titanium alloy plasticity to increase compared with that under low strain rate loadings. Additionally, the stress-strain behaviors of aluminum alloys (AA 5A06 [44], AA 2B06 [45], 2060 [46] and 5A90 [47] aluminum alloy) under high strain rate tensile loading were also conducted. In contrast to reports of the magnitudes of elongation in a wide range of strain rates for titanium alloys, we found that the elongation and ultimate tensile strength of the tested aluminum alloys under high strain rate loading (1.2 × 10^3^~5.0 × 10^3^) were both greatly enhanced, which may be caused by different deformation mechanisms of different materials under the same loading conditions.

**Figure 5:**
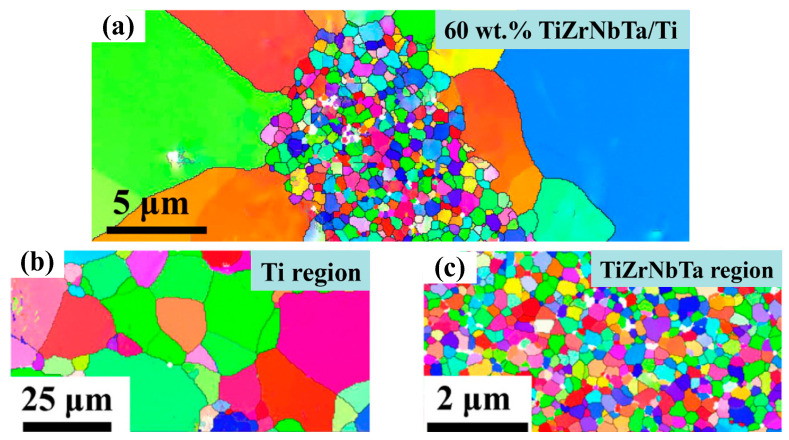
EBSD maps of the optimized TiZrNbTa high entropy alloys/Ti composites (**a**), EBSD maps of Ti-matrix region (**b**) and EBSD maps of TiZrNbTa region (**c**) [51]. Reprinted with permission from Ref. [51]. Copyright 2017, Mater. Sci. Eng. A.

In addition to investigations into the strain rate effect on the elongation and flow stress of titanium alloys, some investigations have focused on improving the grain size structures of materials, such as superplasticity and bimodal grain size distribution (BGSD) [48]. In their examination of superplasticity, Sun et al. found that the decrease in grain size could clearly increase the superplasticity of TC11 alloy [49]. Similarly, Sergueeva et al. revealed that the decrease in grain size in CP-Ti would induce greater hardness and strength compared with as-received specimens [50]. In their investigations of BGSD, Xiang et al. improved the mechanical properties of TiZrNbTa high-entropy alloys/Ti composites by changing the volume fraction of TiZrNbTa in a Ti matrix, as shown in Figure 5 [51]. Experimental results show that the alloys’ yield strength and ultimate strength both reach the maximum value at a mass percentage of TiZrNbTa of up to 60 wt.%. Based on the above results, it can be inferred that the optimization of grain size structure will have a positive effect on improving the service performance of titanium alloy components.

### 2.2. Formability at Room Temperature

**Figure 6:**
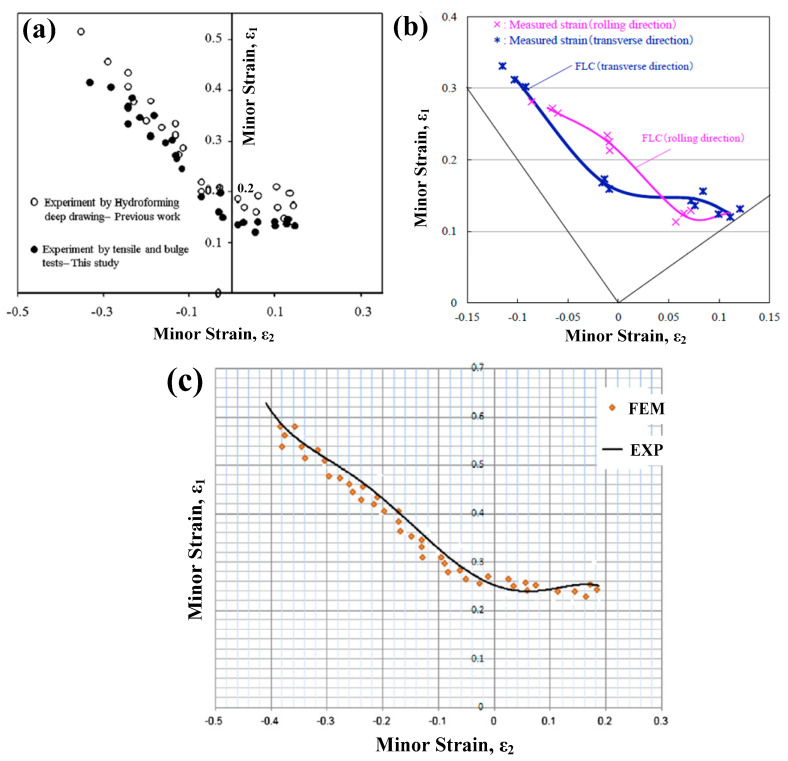
FLD of Ti-6Al-4V alloy sheet obtained by hydroforming at room temperature (**a**) at loaded rate of 1 mm/s [52] (Reprinted with permission from Ref. [52]. Copyright 2019, Results Phys.), (**b**) 1.5 mm/s [54] (Reprinted with permission from Ref. [54]. Copyright 2018, 17th International Conference on Metal Forming.) and (**c**) 1.67 × 10^−3^ mm/s [53] (Reprinted with permission from Ref. [53]. Copyright 2010, Mater. Design.).

The formability of metal sheet is usually determined using a forming limit diagram (FLD). Many scholars have focused on testing the forming limit of titanium alloys under different strain rate loadings at room temperature. Djavanroodi et al. tested the formability of Ti-6Al-4V alloy sheet with hydroforming at room temperature and found that the minimum major strain was less than 0.15 at the loading rate of 1 mm/s according to FLD [52], as shown in Figure 6a. Furthermore, they performed experimental and simulated investigations on the formability of Ti-6Al-4V alloy at a lower loaded rate of 1.67 × 10^−3^ mm/s using hydroforming; the minimum major strain increased to about 0.25 based on the obtained FLD, as shown in Figure 6c [53]. Similarly, Okude et al. conducted experimental tests and simulated predictions of FLD for Ti-6Al-4V alloy sheet with a loaded rate of 1.5 mm/s at room temperature and revealed that the minimum major strain reached 0.21 [54], as shown in Figure 6b. From the above results, we can be inferred that the formability of Ti-6Al-4V alloy will increase at a low strain rate range (1.67 × 10^−3^~1 mm/s). Other than the formability investigations of dual-phase titanium alloys under low strain rate loading at room temperature, many reports have focused on single-dual titanium alloys. Morawiński et al. proposed a novel FLD testing method named analysis of laser speckle activity differences (ALSAD) and tested the formability of grade 1 titanium alloy sheet with a thickness of 0.8 mm [55]. The experimental results show good formability of titanium alloys at the strain level of DC04 stainless-steel (the maximum major strain would reach about 0.5). Yoganjaneyulu et al. studied the formability of titanium grade 2 sheets with single-point incremental forming at room temperature and discovered that the increase in speed and vertical step depth of the forming tools could effectively increase the limiting major true strain value (i.e., the formability could be improved) [56]. By comparing with the formability of α-type titanium and α+β-type titanium alloys, it was found that the limit strain of the former was much larger than the latter at room temperature.

**Figure 7:**
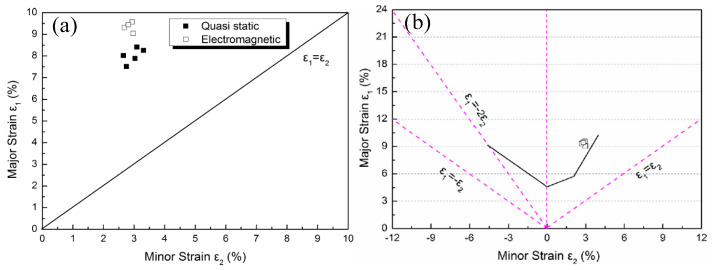
Comparison of formability of Ti-6Al-4V sheets in quasi static (**a**) and EMF (**b**) [57]. Reprinted with permission from Ref. [57]. Copyright 2013, Mater. Design.

Owing to the effect of strain rate, it will certainly show differences in the forming limit at room temperature. However, there are still few reports on recent investigations into titanium alloys’ formability within high strain rate loadings at room temperature. Corresponding formability tests rely on high strain rate forming techniques. Li et al. tested the forming limit of Ti-6Al-4V alloy sheet with electromagnetic forming (EMF) (the electromagnetic force produced by high voltage discharges is induced simultaneously to achieve sheet metal forming) and found that the forming limit increases by 24.37% in a biaxial tension state, as shown in Figure 7 [57]. It was also pointed out that the inertia effect introduced by high strain rate loading changes the fracture behavior of sheet metal, which was the primary reason for the formability improvement of titanium alloys. Additionally, this forming method has also been gradually used in the formability tests on high-strength steel sheet/tubular blanks [58]. Other than EMF, some other high strain rate forming methods, such as electrohydraulic forming (EHF), explosive forming and impact hydroforming (IHF), can be further applied in testing the formability of titanium alloys. However, it has only been found in some recent reports about the formability tests on other materials with high strain rate forming methods mentioned above. To explore the potential applications of these methods on titanium alloys, the forming characteristics of the above high strain rate forming methods are introduced in brief below.

Electrohydraulic forming (EHF) was developed in the 1960s [59]; the forming force of sheet deformation was obtained from the instant release of electrical discharge inside the liquid chamber [60,61,62]. Presently, most experimental investigations are mainly focused on the characterization of the formability of high strength steels, such as DP500, DP590, DP600, DP780, DP980 and others. Yu et al. found that the forming limit height of DP600 sheet increased by 27% with electrohydraulic free bulging compared with that using quasi-static forming and that the contribution of the inertia effect on plastic deformation was 87.1% [63]. Additionally, it was found that the maximum major strain significantly increases under EHF compared with conventional forming. Similarly, Maris et al. compared the forming limits of DP600 and 5182 aluminum alloy sheets with different shapes of tested samples by implementing EHF experiments and found that the principal strain of the two materials increased by 5% and 8%, respectively, compared with that under quasi-static loadings. Further, it was discovered that the formability of the tested materials would greatly improve when the strain rate was over 1000 s^−1^ [64]. Gillard et al. proposed a novel EHF method coupled with quasi-static preforming. The experimental results show that the formability of dual-phase steel sheet (DP780 and DP980) would be further improved with the coupled forming process [65].

In contrast to EMF and EHF, few investigations have been done on the formability of sheet metal on explosive forming or the applications on titanium alloys in the past few decades. The forming principle of explosive forming can be described as being the deformation of sheet metal driven by the impact wave produced by the instance exploding inside air or water [66]. Considering that the explosive tests are dangerous to be performed, many investigations rely on numerical simulations to test the sheet metal’s formability under explosive forming. The formability under explosive forming is relies on the geometric structure of a prefabricated blank before the final forming. Nasiri et al. conducted repeated underwater explosive forming tests with 3 mm thick Armco^®^ iron plates, and 12 g and 4 g PE4 explosive charges were used for single and repeated loading, respectively. According to the experimental and simulated results, smoother thickness distribution and higher formability were obtained with repeated explosive forming [67]. Therefore, it can be concluded that repeated loadings at high strain rates can effectively increase the formability of metal sheet. The formability of titanium alloys under this loading condition warrants further investigated.

**Figure 8:**
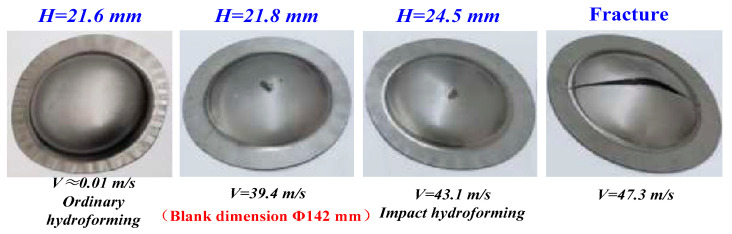
Comparison of the bulging heights of Ti-6Al-4V alloy sheets with different liquid impacting speeds at room temperature [76]. Reprinted with permission from Ref. [76]. Copyright 2021.

Impact hydroforming (IHF) was developed in the 1960s and originally proposed by Bruno [59]. Many scholars have focused on testing the formability of different materials under IHF [68,69]. Akst et al. reported that superior filling level (up to 60%) and more homogeneous hardness distribution along the radial/axial directions could be achieved with components formed under IHF [70]. Niaraki et al. compared the forming limit height of aluminum alloy sheets obtained using IHF and EMF. They found that more uniform deformation of steel sheets occurred in different regions and that the forming height was greatly improved under IHF compared with that under EMF [71]. Hajializadeh et al. experimentally studied the formability of 6061-T6 aluminum alloy thin-walled tubular blanks under IHF and found that the springback of the deformed tubular blank was greatly reduced with the increase in impact energy. Specifically, IHF effectively promotes the material flow from the ends of the tubular blank into the die cavity, which induces the increase in the maximum bulging height of the tubular blank [72]. Our research team also extensively investigated the formability of lightweight alloy sheets with IHF in recent years. Formability tests for 5A06, 2B06 and 2024 aluminum alloy sheets were performed using our self-developed IHF equipment [45,73]. The experimental results showed that the forming limit of the tested aluminum alloys were greatly improved under IHF [74]. Further, we established the quantitative relationships between impact energy and the maximum forming height of aluminum alloys and discovered that the limit deep drawing ratio increased by 5.21% compared with that under conventional deep drawing to 2B06 aluminum alloy. Referring to the established relationship, a thin-walled aviation component with eight deep cavities was obtained with one-stage forming using IHF without fractures [75]. We also conducted a preliminary investigation on the formability of Ti-6Al-4V alloy sheet under IHF. Compared with the maximum bulging height obtained by conventional hydroforming, the ultimate bulging height was basically equal to that under IHF until the loaded impact velocity exceeded 39.4 m/s [76]. Interestingly, as the impact velocity increased to 43.1 m/s, the limit bulging height significantly improved (up to 13.4%), as shown in Figure 8. However, fracture occurred as the impact velocity increased over 47.3 m/s, which illustrates that to achieve good forming quality of titanium alloy components the loaded strain rates need to be reasonably controlled.

In summary, the forming limits of titanium alloy sheet (including other lightweight alloys) at room temperature can be significantly improved under high strain rate forming. Many scholars attribute the formability improvement of titanium alloys to the inertia effect during high strain rate loading. We believe that this phenomenon may have a close relationship with the evolution of micro-structures during high strain rate loading other than that of the inertia effect, which should be further explored in the future experiments.

### 2.3. Deformation Mechanisms of Titanium Alloys

**Figure 9:**
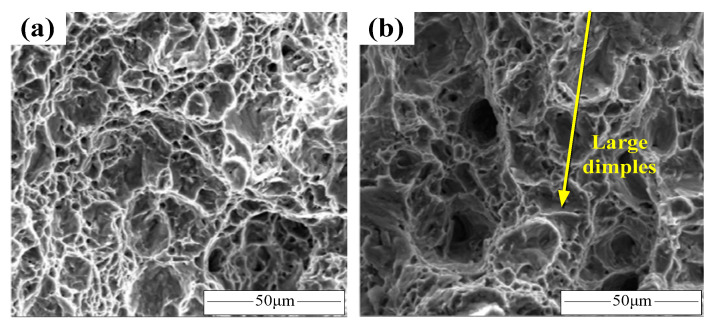
SEM micro-morphology of fracture surface (**a**) 0.01 s^−1^, 2 °C and (**b**) 1500 s^−1^, 25 °C [79]. Reprinted with permission from Ref. [79]. Copyright 2016, J. Alloys Compd.

There are still only a few investigations on the deformation mechanisms of titanium alloy sheet at high strain rate loading at room temperature due to the difficulty of precisely capturing the dynamic deformation process. Current investigations are mainly focused on revealing the potential mechanisms by advanced simulation methods, such as the coupled crystal plasticity finite element method (CPFEM), elasto-plastic self-consistent (EPSC) method, etc. Considering that the plasticity and springback behaviors of sheet metal will be significantly affected by strain hardening and the rate-sensitivity of flow stress, it is necessary to summarize the corresponding analyses on the unique mechanisms of them. In contrast to isomechanical subgroups, α-type titanium alloys have a hexagonal close-packed lattice and the contributions of twinning behaviors on the mechanisms cannot be neglected. Salem et al. discovered that deformation twins would produce hardening of all slip systems and twin systems in a grain [77]. Twin boundaries decrease the slip distance in untwinned regions, which promotes the increase in the strain hardening rate. The saturation of the twin volume faction at a high strain rate loading induces the decrease in strain hardening rate. In addition, the increase in overall strain hardening rate can be explained in that twins are harder than a matrix. Shahba et al. established a crystal plasticity FE model of Ti-7Al alloy for a wide range of strain rates (10^−5^–10^7^ s^−1^) [78]. They found that the rate sensitivity reflected significant change as the strain rate was over 10^5^ s^−1^. Additionally, additional plastic deformation under thermal activation due to adiabatic heating was found at a high strain rate. In addition, many investigations have focused on analyzing plasticity variations by observing the sizes of fracture dimples. For instance, Bobbili et al. discovered that the depth and dimple sizes of the tensile fracture of near-α titanium alloy under high strain rate loading (1500 s^−1^) at room temperature were both much larger than that under a low strain rate loading (0.01 s^−1^) [79], as shown in Figure 9. Based on the above reports, it can be concluded that the hardening behaviors and rate sensitivity of α-type titanium alloys can be attributed to twinning and adiabatic heating under high strain rate loading.

**Figure 10:**
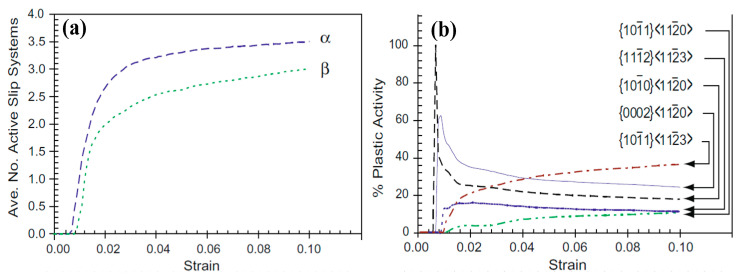

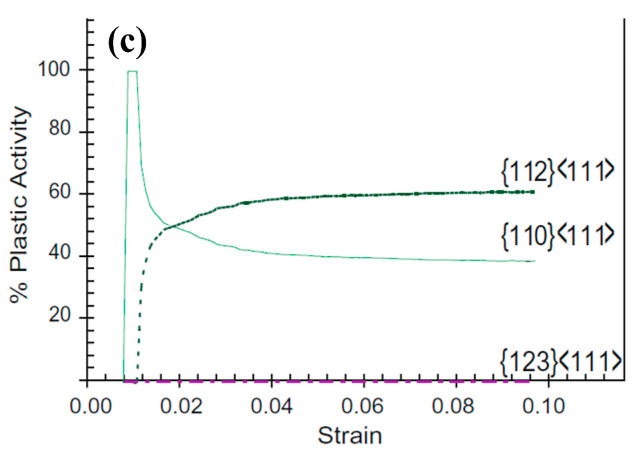
Average number of active slip systems per phase (**a**), relative distribution of active slip systems in the α (**b**) and β (**c**) phases, as predicted by the self-consistent model for the bar [83]. Reprinted with permission from Ref. [83]. Copyright 2008, Acta. Mater.

**Figure 11:**
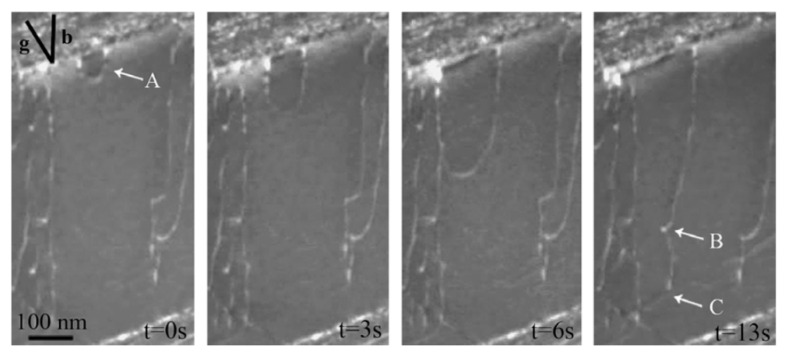
Emission of α dislocation loop from an α_s_/β interface during an in situ TEM observation. (A: the initial position of dislocation emission, B: edge dislocation departs from screw dislocation because of pinning, C: emergence of dislocation loop) [85]. Reprinted with permission from Ref. [85]. Copyright 2007, Acta. Mater.

Regarding the deformation mechanisms of α+β-type titanium alloys (e.g., Ti-6Al-4V alloy) under high strain rate loading, corresponding literatures was not found. Fortunately, many investigations on the deformation mechanisms of titanium alloys are focused on low strain rate loading, which, to some extent, can provide understanding of the deformation mechanism at high strain rate loading. In contrast to α-type titanium alloys, the twinning phenomenon of α+β-type titanium alloys seldomly occurs under low strain rate loading. Therefore, the deformation mechanisms may be significantly different for α-type titanium alloys under high strain rate loading. In general, the analysis of the deformation mechanism of α+β-type titanium alloys at low strain rates involves mainly the starting modes and starting orders of slip systems, the stress-strain co-ordinations of dual-phase interaction, the deformation of grain boundary rotation and the crack of grains [80,81,82]. Stapleton et al. investigated the evolution of lattice strain for Ti-6Al-4V with an EPSC model and discovered that the basal and prismatic slip modes dominated the plastic deformation in the early stage, but the activity of the pyramidal slip modes gradually increased and eventually dominated the plastic deformation in the remaining process for hexagonal α-phase [83]. For the deformation of the β-phase, it was firstly derived from the activity of the {110}<111> slip mode, and then the {112}<111> mode was activated and dominated the deformation in the final deformation stage. The activation modes and the number of slip systems in α- and β-phases are given in Figure 10. Yu et al. investigated the effects of temperature, deformation degrees, strain rates (1.73 × 10^−3^~1.73 × 10^−1^ s^−1^) and their interactions on the microstructure evolution and mechanical properties of Ti-6Al-4V alloy (including equiaxed and lamellar microstructure in the initial state), and found that a high strain rate can make the grain size of the primary α-phase become smaller due to dynamic recrystallization at high temperature (850~980 °C), and would induce an even finer microstructure at higher levels [84]. Castany et al. discovered that the velocity of mobile dislocations of Ti-6Al-4V alloy (the original microstructure includes primary alpha nodules α_P_ and lamellar colonies α_S_/β) was mainly governed by the motion of screw dislocation, and dislocations would firstly emit from the α/β interfaces according to the observed results from in-situ transmission electron microscopy (TEM) experiments [85] (the movement of dislocations during the deformation process is shown in Figure 11). Additionally, basal glide was much easier to activate compared with prismatic glide because of the compatibility stress in the α_s_/β interfaces. In addition to low-energy ratio loadings, electric pulse loading has been recognized as another solution to improve the formability of titanium alloys. Li et al. discovered that electrically-assisted tensile can significantly increase elongation and decrease flow stress compared with room temperature tensile without current [86]. Through further experimental observation, they found that pulse current can improve dislocation movement and decrease dislocation density. Combined with the above discussions, the plastic deformation of α+β titanium alloys originates from the dislocation movement at the α/β interfaces, and the plasticity increase can be attributed to the decrease in dislocation density. Ao et al. discovered that the β-grain was elongated due to dynamic recovery and some α-grains were homogeneously distributed under electropulse loading of Ti-6Al-4V alloy, which can also promote formability improvement [87].

Based on the above reports on the deformation mechanisms of titanium alloys, strain rate will significantly affect the grain sizes after the plastic deformation process of titanium alloys at higher loaded strain rates (lower than 1.73 × 10^−^^1^ s^−^^1^). Furthermore, the sizes of fracture dimples become larger as high strain rates (over 1.5 × 10^3^ s^−^^1^) are applied. It can be inferred that the decrease in dislocation density and increase in dislocation movement may occur under high strain rate loadings, which requires further observation and verification of microstructures in the future.

## 3. Springback Prediction and Control

How to effectively reduce springback of titanium alloy sheet at room temperature is a widely concerning issue in the stamping field because of high strength-to-modulus ratio. The springback behaviors are generally related to the hardening behaviors of sheet metals. Therefore, many scholars have conducted investigations on developing novel yield and hardening models to describe the springback behaviors of titanium alloy sheet in the past few years [88,89,90,91]. The specific discussion about yielding and hardening models of titanium alloys have been conducted in Section 2. In this section, some advanced prediction strategies and control methods of springback for titanium alloys at room temperature are introduced below.

### 3.1. Prediction Methods of Springback Behaviors

**Figure 12:**
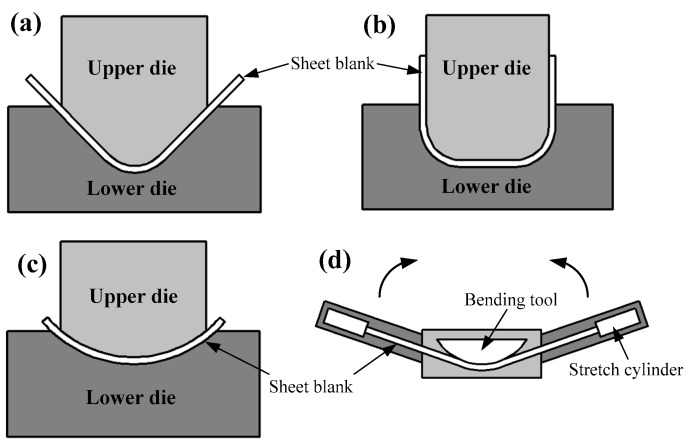
Schematic view of process principles of V-bending (**a**), U-bending (**b**), arc-bending (**c**) and stretch-bending (**d**).

To describe and predict springback behaviors of titanium alloy sheet components with simple geometric shapes at room temperature, analytical analysis is initially considered because of its high efficiency. Currently, analytical analysis is mainly used in springback predictions of simple geometric bending, such as V-bending, U-bending [92], arc-bending and stretch-bending with dual-curved shapes (the process principles of the above bending methods are shown in Figure 12). Previously, Gardiner proposed a springback prediction equation [93] (Equation (1)) for pure bending of elastic and ideal-plastic metal materials. Regarding the effect of material hardening behaviors [94], the Bauschinger effect and potential elastic modulus change [95] are not considered in this model, so it has poor prediction accuracy for springback. Therefore, many scholars have recently revised the equation and proposed novel analytical models considering deformation history [96,97,98]. For instance, Huang et al. proposed an improved equation (Equation (2)) for the elasto-plastic analysis of sheet metal bending considering the variation of plastic modulus on springback and received excellent prediction results compared with experimental results [99]. However, the above models can usually only characterize the shift of the stress-strain neutral layer by analyzing the balance of force moments as it is very difficult to replicate the practical springback behaviors of anisotropic materials.

(1) $$\frac{\rho_{b}}{\rho_{a}}=1-3(\frac{\sigma_{s}\rho_{b}}{Et})+4{\left(\frac{\sigma_{s}\rho_{b}}{Et}\right)}^{3}$$

(2) $$\frac{\rho_{b}}{\rho_{a}}=(1-\frac{D}{E})\left[1-3(\frac{\sigma_{s}\rho_{b}}{Et})+4{\left(\frac{\sigma_{s}\rho_{b}}{Et}\right)}^{3}\right]$$

where *ρ*_a_ and *ρ*_b_ are the radius after and before springback, *D* and *E* are the hardening modulus and the elastic modulus, *σ*_s_ and *t* are the yield strength and the thickness of sheet, respectively.

**Figure 13:**
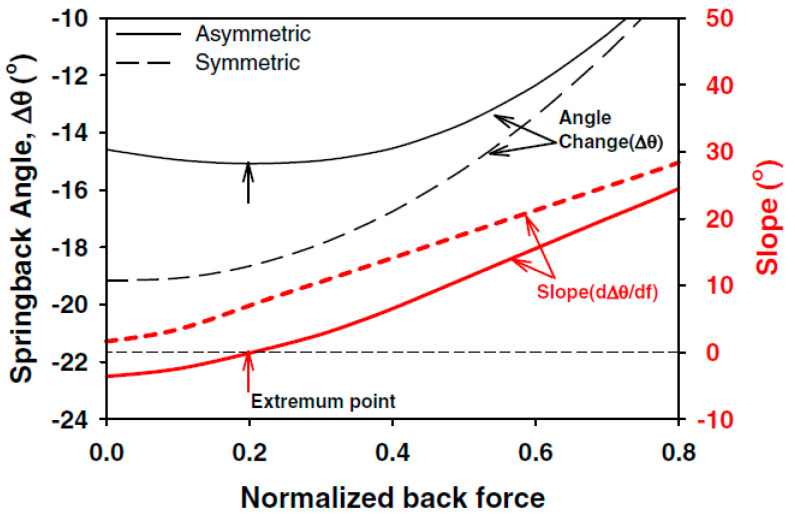
Comparison of calculated springback angles using symmetric and asymmetric models ($\Delta \theta =-\frac{\pi }{2}(1-\frac{R}{r})$, *R* and *r* are the curvature radius before and after springback, *d*_f_ is the variation of normalized back force) [104]. Reprinted with permission from Ref. [104]. Copyright 2009, Int. J. Plasticity.

To predict the springback of anisotropic materials, including titanium alloys, the effect of anisotropy (asymmetry in the yielding and hardening stage) on springback cannot be neglected [100,101]. Zhang et al. found that springback would be overestimated by applying the isotropic hardening model [102]. Nanu et al. found that mechanical anisotropy imposed a significant influence on the stress level of stresses at the inner and outer faces of blanks, which eventually led to more apparent springback [103]. Similarly, Lee et al. proposed an analytical model considering asymmetric elasto-plastic stretch-bending [104]. In comparison to the springback results, it was found that asymmetry would cause an increase in springback of hexagonal close-packed sheet metals compared with using a symmetric material model, as shown in Figure 13.

**Figure 14:**
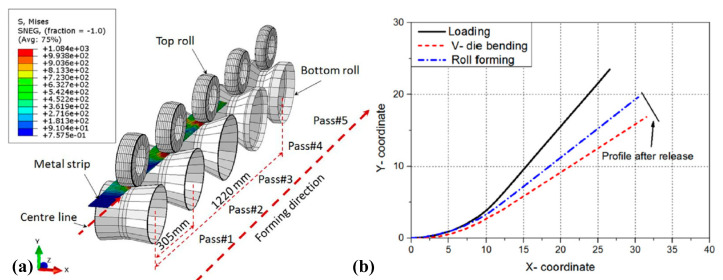
(**a**) Finite element models for V-roll-bending of Ti-6Al-4V sheets and (**b**) springback results obtained by HAH model [107]. Reprinted with permission from Ref. [107]. Copyright 2017, Int. J. Mech. Sci.

Although analytical models have achieved good prediction accuracy for springback of V-bending, U-bending and stretch-bending with simple geometric shapes at low strain rates [105,106], it is still difficult to precisely predict the springback of titanium alloy components with complex geometric shapes under specific loading conditions (e.g., high strain rate loading and multi-stage loading) when relying on only analytical methods. Furthermore, it is also difficult to consider the nonlinear-kinematic hardening behaviors of materials in analytical analyses. In recent investigations, numerical simulation is gradually being developed as an acceptable approach to solve this problem. Badr et al. studied the springback behaviors of Ti-6Al-4V alloy sheet considering cyclic hardening characteristics for V-draw-bending and V-roll-forming developed with a homogeneous yield-function-based anisotropic-hardening model (HAH) model [107] (corresponding finite element models are shown in Figure 14a). Lower springback was found in roll-forming compared with that in draw-bending using forming tools with the same shape and dimensions, as shown in Figure 14b. Moreover, it was verified that the HAH model had higher prediction accuracy of V-bending springback than isotropic-hardening models. In summary, prediction accuracy can be significantly improved by implementing simulation methods combined with advanced hardening models.

### 3.2. Springback Control Methods

Springback control of titanium alloy sheets is achieved by increasing the forming temperature, using advanced the forming/loading methods, compensating the tool surface, etc. Recent investigations have shown that reasonable forming methods may change the microstructure of titanium alloys and further contribute to the reduction in springback. Some advanced springback control strategies for titanium alloys are reviewed below.

#### 3.2.1. Optimization of Loading Paths and Process Parameters

**Figure 15:**
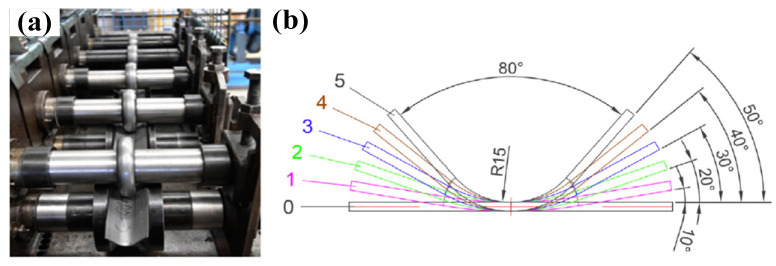

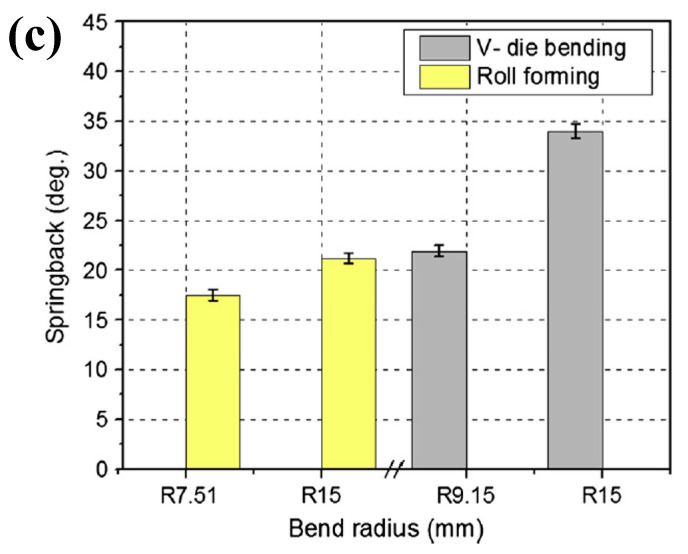
(**a**) Forming tools for roll forming of Ti-6Al-4V sheet, (**b**) flower pattern for the V-profile roll forming with R15 radius and (**c**) springback comparison between V-die-bending and roll forming [112]. Reprinted with permission from Ref. [112]. Copyright 2015, Mater. Design.

Springback of sheet metal can be affected by the loading path and process parameters during the forming process at different strain rates. Therefore, it is necessary to reasonably optimize them to reduce the severe springback of titanium alloys at room temperature. Li et al. decreased the springback of Ti-6Al-4V sheet with an electrically-assisted incremental forming method [108]. In their experimental results, the increase in forming time and pulse current density promoted the reduction in springback at room temperature. Additionally, they discovered that bending cracks could be avoided using pulse current [109]. Odenberger et al. achieved springback reduction in Ti-6Al-4V aerospace engine components with curved surfaces with hot forming. It was found that the optimal forming temperature and the holding time are 700 °C and 150 s, respectively, for springback reduction [110]. In contrast to hot forming, Mo et al. reduced the springback of Ti-6Al-4V alloy curved-surface components with the method of die surface compensation using single-point incremental forming [111]. The designed compensation surface of tools refers to the springback law of the part. Badr et al. applied muti-stage V-roll forming to achieve a tighter profile radius (15% improvement) and less springback (35% improvement) of the Ti-6Al-4V component at room temperature compared with conventional draw-bending [112]. The forming tools and springback results are shown in Figure 15. Leacock et al. investigated the effect of loaded strain rate on the springback of CP-Ti sheet with stretch-bending and found only minor changes in springback at low strain rates of 5.987 × 10^−5^~1.197 × 10^−3^ s^−1^ [113]. Additionally, in order to acquire more accurate and efficient springback prediction of titanium alloys, intelligent algorithms have been used to optimize the forming parameters and loading path in recent reports. For instance, Li et al. applied multi-island genetic algorithm (MGA) optimizing the forming path of cold-drawing of sTC1 titanium alloy aircraft skins, and reduced the springback of the components by 34.98% [91]. Although the above investigations can effectively reduce the springback of titanium alloys, the efficiency of them still needs to be improved.

**Figure 16:**
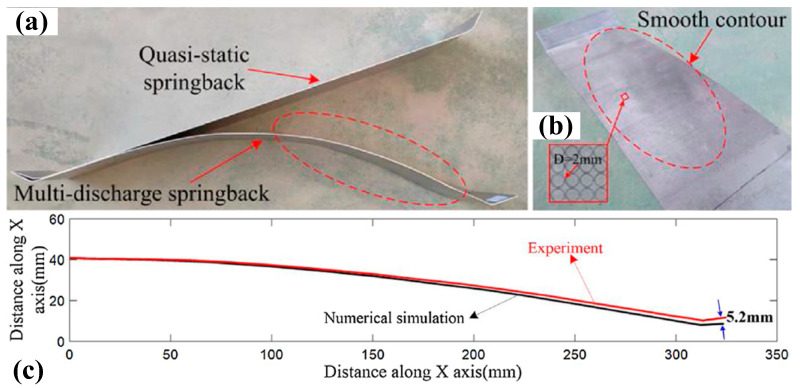
Springback results of 3033 aluminum alloy sheets obtained by conventional stretching and electromagnetic partitioning forming [12]. Reprinted with permission from Ref. [12]. Copyright 2022, J. Mater. Process. Tech. (**a**) Quasi-static and multi-discharge springback, (**b**) surface contour of obtained arc part and (**c**) comparison of the cross-section profiles of experiment and numerical simulation.

**Figure 17:**
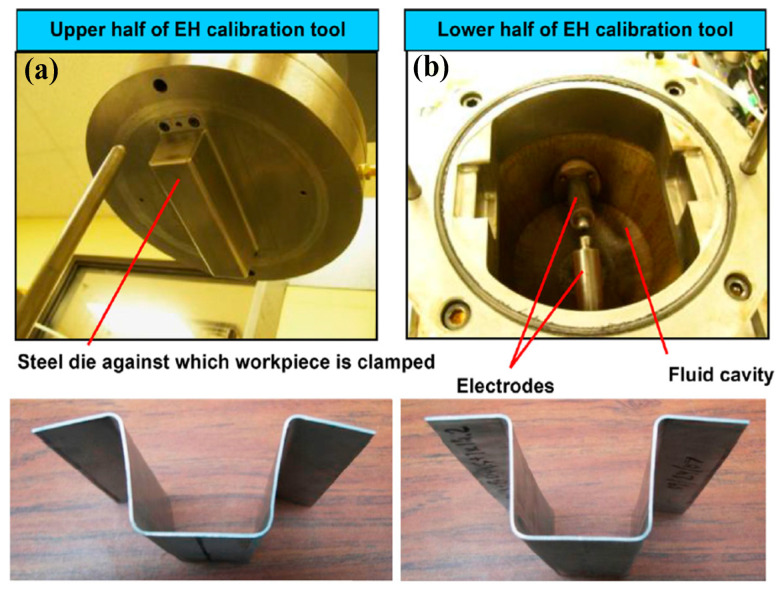
Springback calibration of 6111-T4 aluminum sheet with EHF [115]. Reprinted with permission from Ref. [115]. Copyright 2014, J. Mater. Process. Tech. (**a**) Before calibration and (**b**) after calibration.

Presently, springback calibration technology with high strain rate loading has been proposed, and the level of springback reduction has also been tested with aluminum alloys and stainless-steels. Cui et al. realized springback reduction in 3033 aluminum alloy and 304 stainless-steel skin-shaped components with electromagnetic-partitioning-forming [12,114], the experimental results of which are shown in Figure 16. Based on their simulation results, electromagnetic partitioning-forming changes the stress state of the local region of sheet blanks by introducing high-speed oscillating stress-waves compared with conventional deep drawing, which produces larger plastic deformation and eventually realizes a reduction in springback. Golovashchenko et al. conducted springback calibrations of 6111-T4 aluminum sheet with pulse electrohydraulic-forming [115] and found that springback could be significantly reduced under pulse pressure loading; the obtained experimental results are displayed in Figure 17. By analyzing the simulation results, the main reason for springback reduction was due to the relief of internal stress. Overall, high strain rate loading is mainly used to conduct springback calibration at room temperature in the current investigations.

**Figure 18:**
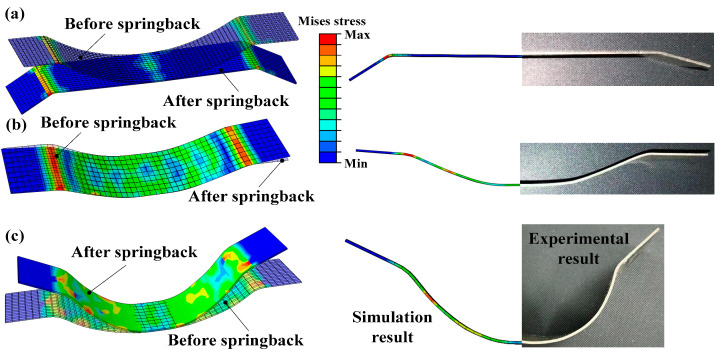
Simulated and experimental springback results of Ti-6Al-4V arc-bent parts under IHF [116]. Reprinted with permission from Ref. [116]. Copyright 2022, International Deep-Drawing Research Group Conference. (**a**) Conventional arc-bending with velocity of 0.1 m/s, (**b**) IHF with velocity of 43 m/s and (**c**) IHF with velocity of 65 m/s.

Similar to the springback calibration under high strain rate loadings, we also carried out a preliminary study on the springback reduction in Ti-6Al-4V alloy sheet arc-bending with self-developed impact hydroforming [116]. The experimental results showed that with conventional bending as shown in Figure 18a, severe springback occurs after unloading. Interestingly, the springback of titanium alloy sheets significantly decreases under IHF, and it is even eliminated as the loaded impact velocity is controlled within a reasonable threshold value (43 m/s), as shown in Figure 18b. Remarkably, with the loaded strain rate increasing to 65 m/s, the titanium alloy sheets incur negative springback, as shown in Figure 18c. However, the potential mechanism for springback reduction has not been revealed and requires further investigation.

#### 3.2.2. Mechanisms of Springback Induction and Reduction

**Figure 19:**
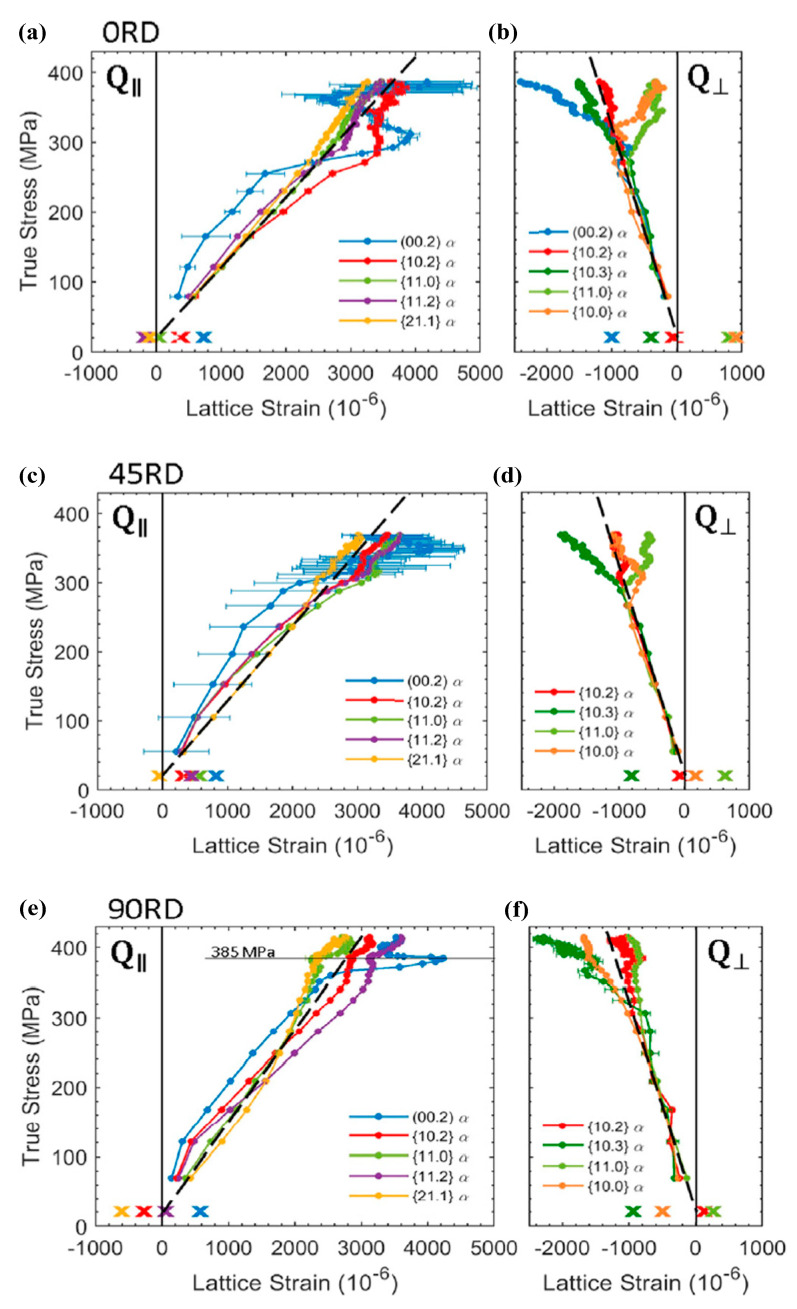
Lattice strain evolution versus applied stress for (**a**,**b**) 0RD, (**c**,**d**) 45RD and (**e**,**f**) 90RD specimens. Selected axial (Q_II_) and radial (Q_⊥_) α-grain families are shown on the left and right, respectively. The unload of the lattice strain is linear for all grain families and is not shown for clarity. The residual lattice strains after unload are marked by “x” [117]. Reprinted with permission from Ref. [117]. Copyright 2019, Acta. Mater.

Currently, there are still few investigations on the mechanisms of the springback induction and springback reduction in titanium alloys. Considering that titanium alloys belong to the hexagonal close-packed metals, the distinctive springback behaviors in different loading directions should be considered. Sofinowski et al. investigated the effect of hardening behaviors on the springback of grade 2 α-titanium in three loading directions (RD, DD and TD) with situ XRD and quasi-in situ EBSD and HRDIC and discovered that three hardening stages were exhibited in the tensile process and a non-linear anelastic springback occurred in the unloading stage [117,118]. The non-linear phenomenon of springback was for the annihilation/rearrangement of dislocations caused by the backforce. Further, it was found that springback in the RD direction was significantly larger than that in other loading directions [119], which was attributed to the larger residual strain of the radial α-grain families in other loaded directions, as shown in Figure 19. Zhao et al. investigated the springback characteristics of Ti-29Nb-13Ta-4.6Zr (TNTZ) and discovered that the deformation-induced phase in Ti-12Cr could decrease springback compared with that of TNTZ without the deformation-induced phase [120]. It inferred that the induced phase of Ti-based biomaterials in deformation could promote the springback reduction. Ao et al. investigated the effect of electropulsing loading on springback reduction in Ti-6Al-4V sheet during the V-bending process. They stated that the clustered β-phase would be dissipated under electropulsing loading, resulting in eradication of the piled-up dislocations surrounding β-phase and thus springback reduction [11,121]. Considering that magnesium alloys have a similar crystal structure (including hexagonal close-packed phase) to titanium alloys, the potential mechanisms of springback reduction will have something in common with that of titanium alloys. Wang et al. tested the effect of forming temperature and punch radius on the evolution of the microstructure and the shift of the neutral layer of AZ31 magnesium alloy sheet during V-bending, and found that the {1,0,−1,2} tensile-twinning dominated the deformation compression inner, while the slip dominated the tension outer [122]. It should be noted that the tension-compression asymmetry in inner and outer fiber layers became weaker with the increase in temperature. Additionally, in comparison to isotropic materials in the tensile zone, the neutral fiber layer shifted to the outer fiber layer with the decrease in punch radius. Therefore, it can be inferred that the springback reduction in titanium alloy has a close relationship with the shift of the neutral layer and the weakness of the tension-compression asymmetry due to the evolution of the microstructure.

According to the above investigation reports, reducing the springback of titanium alloy sheet at room temperature is mainly through increasing the plastic strain and relieving the residual strain by improving the forming processes. It should be noted that high strain rate loading can be effectively achieved in springback reduction in titanium alloys at room temperature. However, current investigations regarding this point are mainly on the springback calibration of aluminum alloy and stainless-steel sheets, whereas similar reports of titanium alloys are not available. In our own investigations, the severe springback of titanium alloys at room temperature can be dramatically reduced under the self-developed IHF method, as shown in Figure 16. The potential mechanisms for springback reduction warrant further investigation.

## 4. Summary and Perspective

Many conventional and advanced methods to improve formability and reduce springback in titanium alloy sheet at room temperature are systematically reviewed in this paper. The methods for formability improvement include elevating forming temperature (e.g., superplastic forming and electropulsing forming) and controlling the loaded strain rates at extremely low or high levels. On the other hand, the methods for springback reduction are involved in compensating the tool surface, optimizing the loading path, increasing the loaded strain rates, etc. Currently, investigations on the deformation mechanisms of titanium alloys are mainly focused on low strain rate loadings (below 0.1 s^−1^), and the findings of the potential mechanisms are focused on dislocation evolution and phase distribution observed using OM, SEM, TEM and EBSD. Regarding the springback prediction of titanium alloys at room temperature, the effect of asymmetry cannot be neglected. To realize springback reduction in titanium alloys, improving the amount of plastic deformation and making microstructures more uniform in different fiber layers are considered acceptable solutions at room temperature.

Based on recent investigations and our preliminary studies, the formability of titanium alloy parts can be improved and the produced springback will be reduced under high strain rate loading. The developed forming methods for springback reduction in lightweight alloys at high strain rates include electromagnetic pulsing calibration, electro-hydraulic loading calibration and impact hydroforming. Specifically, impact hydroforming has been tested and achieved springback reduction in titanium alloys with a one-stage forming process. According to recent results, the increase in formability of titanium alloy components under high strain rate loading contributes to the inertia effectiveness. However, the mechanism of the strain rate effect on formability improvement and springback reduction is still not fully understood. Further investigation into the mechanisms are still required, and are suggested as follows:

(1) Explore the plastic deformation mechanisms of titanium alloys at room temperature, especially under high strain rate loadings. The further micro-scale experimental observations should be conducted with advanced experimental measurement methods (HR-EBSD, in-situ EBSD, in-situ TEM, etc.) and advanced simulation approaches (VPSC, EPSC, CPFEM, etc.). Careful observations should be aimed at the activated modes and orders of the slip systems, deformation coordination and interaction mechanism of dual-phase structures, deformation of the grain boundary, etc. In addition, the variations in dislocation density and storage energy during high strain rate forming process should be calculated to show the potential mechanism for springback reduction in titanium alloy sheets.

(2) The evolution laws of in-plane anisotropy of titanium alloys under high strain rate loadings warrant further investigation. The threshold value of the strain rate for the plasticity increase in titanium alloys should be determined. The quantitative characterization of the inertia effect for the formability improvement of titanium alloys under high strain rate loadings should be further performed. On the other hand, it is also necessary to calculate the contribution ratio of microstructure evolution on the formability improvement of titanium alloys. Additionally, the optimization of forming parameters should be implemented to simultaneously balance the formability improvement and springback reduction.

(3) Analytical analysis of springback reduction under high strain rate loadings should be conducted. The analytical calculation should include the shift of the stress-strain neutral layers in the thickness direction. Furthermore, the quantitative characterization of the distributions of the stress state and plastic strain in the thickness direction at high strain rate loadings should also be performed via simulations.

As perspective, solving the forming difficulties of titanium alloys at room temperature is meaningful in improving both the manufacturing precision and efficiency in practical production. Especially for high strain rate forming, it can further increase the production efficiency of titanium alloy components in future applications. In addition, more advanced simulation technologies, observation methods for microstructure evolution and intelligent algorithms will be developed and applied to characterize the deformation behaviors of titanium alloys at macro- and micro-scales at room temperature. Further, the developed methods for improving the forming precision of titanium alloys at room temperature may be utilized to solve the forming difficulties of other lightweight alloys in the future.

## Data Availability

Data sharing not applicable.
